# Application of Adipose Extracellular Matrix and Reduced Graphene Oxide Nanocomposites for Spinal Cord Injury Repair

**DOI:** 10.1002/adhm.202402775

**Published:** 2024-12-12

**Authors:** Kest Verstappen, Lara Bieler, Nathalie Barroca, Ewald M. Bronkhorst, Sébastien Couillard‐Després, Sander C.G. Leeuwenburgh, Paula A.A.P. Marques, Alexey Klymov, X. Frank Walboomers

**Affiliations:** ^1^ Department of Dentistry‐Regenerative Biomaterials Radboud University Medical Center Nijmegen 6525 EX The Netherlands; ^2^ Institute of Experimental Neuroregeneration Paracelsus Medical University Salzburg 5020 Austria; ^3^ Austrian Cluster for Tissue Regeneration Vienna 1200 Austria; ^4^ Centre for Mechanical Technology and Automation (TEMA) Intelligent Systems Associate Laboratory (LASI) Department of Mechanical Engineering University of Aveiro Aveiro 3810‐193 Portugal

**Keywords:** biomaterials, extracellular matrix, graphene, nanocomposite, regeneration, spinal cord injury

## Abstract

Graphene‐based materials (GBMs) hold strong promise to restore the spinal cord microenvironment and promote functional recovery after spinal cord injury (SCI). Nanocomposites consisting of reduced graphene oxide (rGO) and adipose tissue‐derived extracellular matrix (adECM) are known to promote neuronal growth in vitro and to evoke a biocompatible response in vivo when implanted on top of the intact spinal cord. In this study, pristine adECM and adECM‐rGO nanocomposites are implanted directly after hemisection SCI in rats. Scaffolds composed of collagen type I (COL) are applied as negative control, based on evidence that COL triggers integrin‐mediated astrogliosis. However, COL scaffolds induce orthotopic bone formation in the lesion site and are therefore excluded from further analyses. Compared to pristine adECM, adECM‐rGO nanocomposites completely restore spinal cord integrity. Macrophage‐mediated uptake and clearance of rGO remnants is observed as early as 3 weeks post‐implantation. Nanocomposites show an elevated presence of βIII‐tubulin‐positive axons in the host‐material interface after 8 weeks, yet scaffold penetration by axons is only occasionally observed. This is partially due to an increased expression of chondroitin sulfate proteoglycans (CSPGs) within the nanocomposites, even though reactive astrogliosis is unaltered. Despite the complete restoration of tissue architecture, adECM‐rGO treatment does not significantly improve functional recovery.

## Graphical abstract

Implantation of nanocomposites, composed of adipose tissue‐derived extracellular matrix (adECM) and reduced graphene oxide (rGO), results in a complete restoration of the integrity of the injured spinal cord. The presence of axons within the host‐nanocomposite interface is increased and myelinated axons are observed to infiltrate the scaffold, while remnants of rGO are being cleared from the lesion site by macrophages. Created with BioRender.com

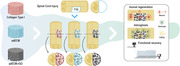

## 1 Introduction

Spinal cord injury (SCI) is a catastrophic insult to the central nervous system (CNS) that results in partial or complete loss of motor and sensory function below the injury site. The often permanent neurological impairment confronts affected individuals with devastating physical, social, and vocational consequences.^[^ ^1^, ^2^ ^]^ The initial trauma permeabilizes neurons and glia, damages the vasculature, and disrupts the blood‐brain barrier. Consequently, ischemia, inflammation, and progressive cell death all contribute toward a detrimental secondary injury cascade.^[^ ^3^ ^]^ Resident astrocytes become activated and hypertrophic in a process called astrogliosis, proliferate and subsequently migrate toward the site of injury.^[^ ^4^ ^]^ Here, reactive astrocytes combine with extracellular matrix (ECM) proteins that are inhibitory toward axonal growth, such as chondroitin sulfate proteoglycans (CSPGs), to form a cellular and biochemical mesh‐like barrier called the glial scar.^[^ ^3^, ^5^ ^]^ Although scarring restricts initial injury progression, remodeling of the lesion eventually establishes a microenvironment, hallmarked by cystic cavities and astrogliosis, that potently inhibits endogenous as well as *de novo* neurite outgrowth.^[^ ^5^, ^6^ ^]^ Biomaterial therapies hold strong promise to facilitate repair of the injured spinal cord, and could be applied to i) restore tissue architecture, ii) counteract the inhibitory microenvironment hence aid in cell survival and differentiation of both endogenous and transplanted cells, and iii) facilitate the directed outgrowth of axons.^[^ ^6^, ^7^ ^]^ Nevertheless, leading‐edge clinical evidence reports only incremental success,^[^ ^8^ ^]^ thereby reflecting the complex pathophysiology of SCI and adding up toward the immense need for advanced biomaterials to treat this condition.

Graphene‐based materials (GBMs) have emerged as a promising class of biomaterials to target nervous system disorders, such as SCI.^[^ ^9^, ^10^ ^]^ The specific morphological, electrical, and mechanical properties of graphene and its derivatives have been reported to promote stem cell differentiation and neuronal growth.^[^ ^11^, ^12^ ^]^ For instance, graphene nanoribbons are suitable morphologies for neural differentiation and are known surface templates for osteogenesis as well.^[^ ^13^, ^14^ ^]^ Importantly, increased neuronal differentiation was partially attributed to mechano‐transduction pathways altered by the local mechanical properties of reduced graphene oxide (rGO).^[^ ^15^ ^]^ Pioneering preclinical applications of rGO scaffolds prevented the formation of cystic cavities, thereby showing the material could serve as a biocompatible neuronal interface following SCI.^[^ ^16^, ^17^ ^]^ In the longer term, implantation of mechanically compliant rGO foams led to immunomodulatory effects, promoted angiogenesis, and demonstrated the presence of axons both within the scaffold and surroundings of the lesion site.^[^ ^18^, ^19^ ^]^ Changing the rGO topography^[^ ^20^ ^]^ or immobilizing growth factors onto PLGA‐GO nanofibers^[^ ^21^ ^]^ further increased the presence of neuronal markers at the injury site, but did not result in improved functional recovery. In contrast, the initial application of organic‐inorganic chitosan‐GO nanocomposites following complete SCI promoted functional recovery, although the specific histomorphometric analyses failed to justify these effects.^[^ ^22^, ^23^ ^]^ In very recent evidence, implantation of amino‐functionalized graphene, incorporated into collagen type I scaffolds, stimulated ECM remodeling and axon regeneration and consequently promoted functional recovery as early as 14 days post‐SCI.^[^ ^24^ ^]^ Altogether, these results indicate the benefit of GBMs, especially when integrated into naturally derived materials, to treat SCI.

Such naturally derived biomaterials are particularly promising for regenerative medicine applications owing to their inherent bioactivity and native ligand biodistribution.^[^ ^6^ ^]^ Ample evidence exists showing that implantation of decellularized extracellular matrices (dECM), formed by removing immunogenic cellular components from native tissues,^[^ ^25^ ^]^ alleviated inflammation, stimulated axonal regeneration, and promoted functional recovery following SCI.^[^ ^26^, ^27^ ^]^ To this end, a nanocomposite biomaterial was recently developed which consisted of rGO and ECM derived from adipose tissue (adECM), since neurotrophic properties do not seem to be related to tissue specificity of the matrix source.^[^ ^28^, ^29^ ^]^ Moreover, accessibility of the matrix source is a major consideration when developing biomaterial therapeutics.^[^ ^30^ ^]^ Compared to alternative sources, adipose tissue is more readily available, less restricted by ethical constraints, and more suitable for manufacturing scale‐up toward future applications.^[^ ^31^, ^32^ ^]^ The specific adECM used here still preserves many of the native proteins that are indispensable for tissue regeneration, such as laminin and fibronectin, and adECM‐derived hydrogels previously exhibited anti‐inflammatory properties.^[^ ^33^, ^34^, ^35^, ^36^, ^37^ ^]^ Importantly, recently developed adECM‐rGO composites promoted neuronal differentiation, and embryonic progenitors developed longer and thicker axons.^[^ ^38^ ^]^ In addition, a recent safety evaluation, which involved scaffold implantation on top of an intact spinal cord, demonstrated that adECM‐rGO composites evoked a biocompatible, pro‐regenerative, and angiogenic response along with the absence of any systemic toxicity.^[^ ^39^ ^]^ Summarizing, the regenerative features of GBMs and naturally derived materials, along with the safe application of adECM‐rGO composites, encourage the application of graphene‐containing nanocomposites in an experimental model of SCI.

Therefore, a right‐sided hemisection was introduced in the current study at the 10^th^ thoracic vertebra (T10) in rats followed by the implantation of adECM and adECM‐rGO scaffolds into the lesion site. In addition, scaffolds composed of collagen type I (COL) were included to serve as a negative control, based on observations that demonstrated a detrimental COL‐mediated effect on astrogliosis which subsequently hampered functional recovery following SCI.^[^ ^40^ ^]^ Spinal cord tissue was examined 1, 3, 5, and 8 weeks post‐SCI. Axonal outgrowth, astrocyte reactivity, and expression of CSPGs were evaluated 8 weeks post‐SCI. Functional recovery of all rats was monitored before, and 3 days as well as 1, 4, and 8 weeks post‐SCI.

## 2 Results and Discussion

There is an urgent need for advanced biomaterial‐based therapies for SCI, capable of i) restoring tissue architecture, ii) counteracting on the inhibitory microenvironment to allow infiltration as well as differentiation of endogenous cells, and iii) serving as guidance to direct outgrowth of regenerating axons. In this context, an adECM‐rGO nanocomposite was developed, which previously guided neuronal differentiation and growth in vitro^[^ ^38^ ^]^ and evoked a biocompatible response in vivo.^[^ ^39^ ^]^ To determine the regenerative potential of this promising nanocomposite in an experimental model of SCI, the current study evaluated the restoration of spinal cord integrity, potential axonal growth and astrogliosis, and recovery of locomotor function. For clarification, in this work, GO was submitted to discrete reduction at low temperatures and for a short period of time to retain oxygen moieties and facilitate potential coupling with natural proteins. In these conditions, the rGO used in this work is characterized by the following properties: i) C/O ratio of 2.85, with 35.1% of oxygen functionalities, 62.5% of C─H and C═C bonds, and 2.4% of π–π^*^ transitions;^[^ ^41^ ^]^ ii) carbon lattice with residual structural stress and disorder as reflected in Raman spectroscopy by both an I_D_/I_G_ ratio of 1.72 (as opposed to ratio of 1.6 for GO) and a lowered position of the 2D band at 2657 cm^−1^ (in relation to GO at 2682 cm^−1^);^[^ ^42^, ^43^ ^]^ iii) zeta potential of ≈−26.6 ± 2.1 mV comparing to pristine GO (−35.2 ± 2.7 mV); and iv) lateral dimensions greater than 6 µm resulting from the reduction‐induced stacking of multiple crumpled and/or folded sheets (as analyzed by AFM).^[^ ^41^ ^]^

### 2.1 High Survival Rates and Unaltered Animal Welfare Following SCI and Implantation

The severity of preclinical SCI requires a meticulous model execution and monitoring of animal welfare.^[^ ^44^ ^]^ Figure S1A (Supporting Information) shows that injured animals suffered from an initial drop in weight. The majority of these rats, however, had reached the starting weight at the end of the experimental period. Importantly, no differences were observed between the COL, adECM, and adECM‐rGO treatment groups. Animal survival (Figures S1B,C, Supporting Information) was generally observed to be in line with similar studies.^[^ ^45^ ^]^ However, a fraction of animals was prematurely excluded (*n* = 3) due to a lack of recovery in urinary function. Interestingly, this set of animals all received an adECM‐rGO implant, suggesting a scaffold‐mediated effect on urinary function. However, neither systemic toxicity nor organ‐specific damage or inflammation in kidney and bladder tissue was observed following previous implantation of similar nanocomposites.^[^ ^39^ ^]^ Nevertheless, when immersed in PBS, adECM‐rGO composites demonstrated increased swelling at equilibrium compared to their adECM counterparts. Although unlikely, implantation into the injury site could have resulted in compression of intact, contralateral tissue which consequently could have affected urinary function.

### 2.2 Tissue‐Restorative and Angiogenic Response following Scaffold Implantation

**Figure 1:**
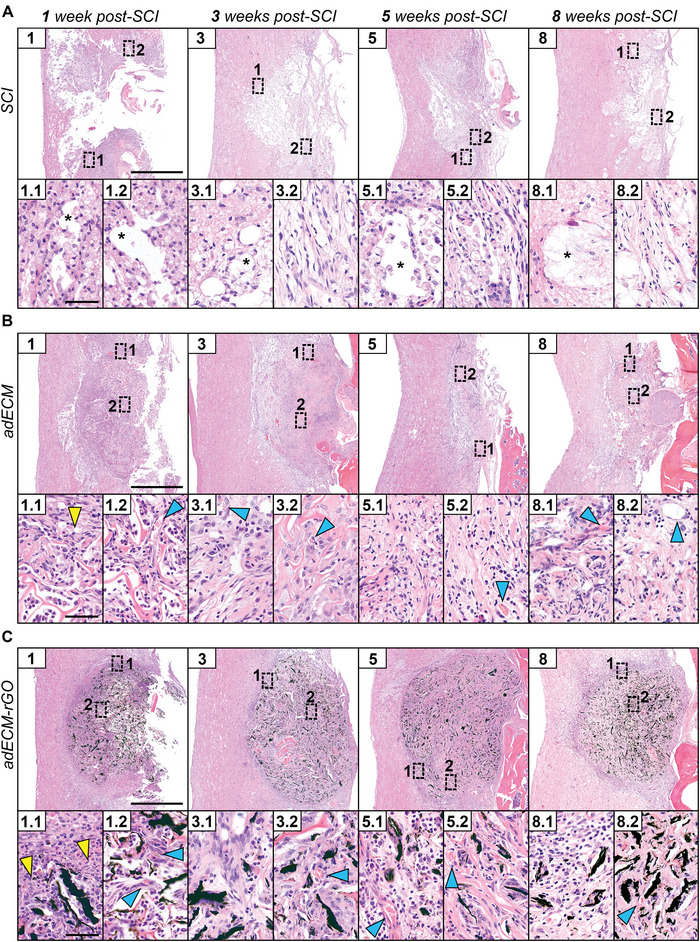
Restoration of tissue architecture and ingrowth of cells, tissue, and blood vessels following adECM and adECM‐rGO implantation. H&E histological staining was performed to assess the spinal cord tissue in the SCI (A), adECM (B), and adECM‐rGO (C) groups (from top to bottom) at 1, 3, 5, and 8 weeks post‐SCI (from left to right). For each condition and timepoint, two inserts have been included that contain higher magnification images of the peripheral (1) or central (2) regions of the lesion site. Stars represent cavitations, yellow arrows depict the direction of tissue ingrowth 1‐week post‐implantation, and blue arrows demonstrate the presence of blood vessels. Note that for SCI, the lesion remodeling is hallmarked by the formation of cystic cavitations, in A(1–8).1, and deposition of loose connective tissue, in A(3–8).2. Ingrowth of tissue (1.1) and blood vessels, in B(1–8).2, is observed as early as 1‐week post‐implantation of adECM scaffolds. Similarly, adECM‐rGO demonstrates the presence of tissue ingrowth (C1.1) and blood vessels, in C(1–8).2, which are in close contact with rGO (black). Scale bars represent 1000 µm (overviews), or 50 µm (inserts).

**Figure** **1** shows the injury control, adECM, and adECM‐rGO treatment groups following 1, 3, 5, and 8 weeks post‐SCI. The damage inflicted by hemisection is represented by a completely disorganized connective tissue 1 week after injury (Figure 1A1). Contrasting the injury control, implantation of adECM and adECM‐rGO seemed to restore the overall integrity of the cord and allowed for a significant cellular infiltration (Figures 1B,C), which further stabilized the implants in line with previous reports.^[^ ^18^ ^]^ As early as 1 week post‐injury, adECM scaffolds were observed to be completely infiltrated (Figure 1B1.2), whereas ongoing gradual cellular infiltration was seen in their graphene‐based counterparts (Figure 1C1.2). Nevertheless, after 3 weeks of implantation, full colonization by infiltrating cells, as well as matrix ingrowth, was observed, hence fulfilling the criterion of guided tissue growth as a prerequisite for an effective biomaterial.^[^ ^46^ ^]^ Over time, the formation of cystic cavities (Figure 1A.1) and deposition of loose connective tissue (Figure 1A.2) hallmarked lesion remodeling in the empty control, while adECM and adECM‐rGO scaffolds integrated well into adjacent, surrounding spinal cord tissue. After 8 weeks, implantation of both adECM and adECM‐rGO significantly limited the presence of cystic cavities (Figure S2A, Supporting Information), which are known to be a physical barrier for axonal outgrowth.^[^ ^47^ ^]^ Various similar applications of GBMs upon SCI have not reported such values,^[^ ^18^, ^20^, ^21^, ^22^, ^23^ ^]^ whereas both adECM and adECM‐rGO scaffolds highly likely restricted extensive tissue necrosis and thereby eventual cyst formation, indicative of strong tissue integration. Unlike their graphene‐based counterparts, adECM scaffolds additionally integrated with more dorsally located cartilaginous and bony tissue of the vertebral column (Figure 1B8), which can be observed in similar reports.^[^ ^28^ ^]^ Future investigations should undertake preventative measures, such as dural closure upon hemisection, that limit the interaction between the lesion and surrounding tissues.^[^ ^48^ ^]^

Besides the loss of tissue integrity, restoration of the dysfunctional neurovascular microenvironment is one of the major challenges following SCI.^[^ ^49^ ^]^ Angiogenesis is required for the diffusion of oxygen and supply of nutrients, both essential for any successful tissue regeneration to take place, but is also closely intertwined with subsequent neurogenesis.^[^ ^50^, ^51^ ^]^ Indeed, the application of an angiogenic hydrogel following stroke induced the formation of a vascular bed in the cavity and patterned axonal growth along its vessels.^[^ ^52^ ^]^ In the current study, *de novo* formation of blood vessels was observed for both adECM and adECM‐rGO scaffolds as early as 1 week post‐implantation (Figures 1B1.2,C1.2), which could be observed throughout the experimental period. After 8 weeks of implantation, blood vessels were detected even in the most centrally located regions of both types of scaffolds (Figures 1B8.2,C8.2), corroborating the strong angiogenic properties that were previously reported for both ECM‐based materials as well as GBMs.^[^ ^19^, ^26^ ^]^

### 2.3 Evidence of Macrophage‐Mediated Uptake of rGO

**Figure 2:**
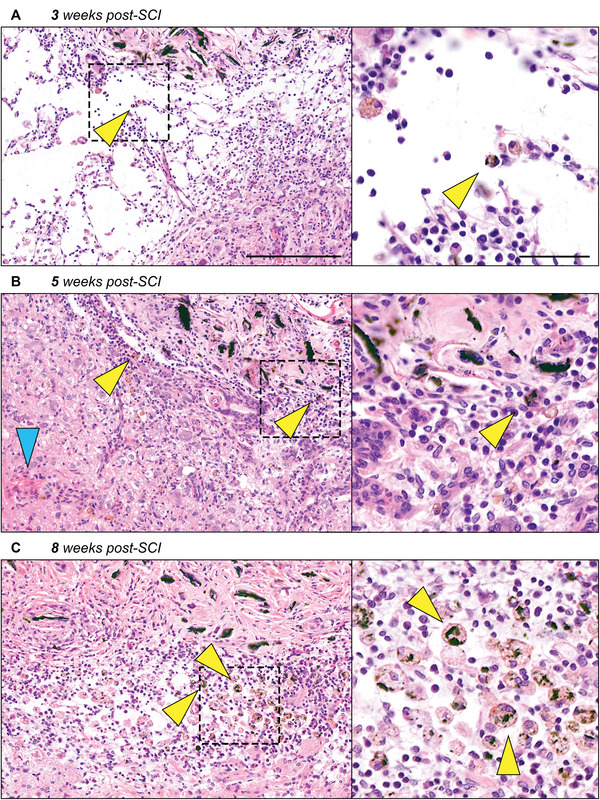
Macrophage‐mediated cellular uptake of rGO remnants as early as 3 weeks post‐implantation. H&E histological staining was performed to assess the potential clearing of rGO sheets from the lesion site at 3, 5, and 8 weeks post‐SCI (from top to bottom). For each time point, one insert has been included that contains higher magnification images demonstrating the intracellular presence of rGO (black) remnants. Yellow arrows depict the intracellular presence of rGO, and blue arrows depict nearby blood vessels from which phagocytic cells highly likely originated. The host‐material interface demonstrated a strong presence of macrophage‐mediated uptake as early as 3 weeks post‐implantation (A) and was more clearly present after 5 (B) and 8 weeks (C) after implantation. Scale bars represent 1000 µm (left), or 50 µm (right).

Understanding the biodegradability of rGO upon implantation is crucial when evaluating whether this material is a suitable component for therapeutic biomaterials.^[^ ^53^ ^]^ Previous research demonstrated the engulfment of rGO by foreign body giant cells (FBGCs), along with indications of ongoing degradation.^[^ ^39^ ^]^ However, no insight into the temporal distribution of macrophage‐mediated phagocytosis was provided. In the current study, active cellular uptake of rGO fragments by macrophages could be observed as early as 3 weeks post‐SCI (**Figure** **2**A). Macrophages were present close to nearby blood vessels (Figure 2B) and populated the host‐material interface. In these regions, there is clear evidence that rGO remnants were being internalized by macrophages, up to 8 weeks after implantation (Figure 2C). Likewise, graphene has previously been shown to activate the phagocytic system which resulted in its uptake and subsequent biodegradation by macrophages.^[^ ^54^, ^55^ ^]^ Specifically, oxidative enzymes were shown to inflict structural disorders on the material following engulfment, which resulted in complete amorphization of graphene after 3 months.^[^ ^54^ ^]^ The engulfment of rGO by FBGCs as well as the consistent attraction of macrophages to the host‐material interface observed in this study shows the clearance of rGO from the lesion site. Since bio‐persistent rGO potentially evokes chronic inflammation,^[^ ^56^ ^]^ eventual clearance is an invaluable requirement for the safe application of GBMs as SCI treatment. Given this, future investigations should verify the long‐term metabolic process of rGO in the body and its biological toxicity after macrophage uptake. The biocompatibility of rGO‐modified adECM nanocomposites was recently investigated, yet only after 6 weeks of implantation.^[^ ^39^ ^]^ In this previous study, biochemical plasma analysis indicated systemic compatibility and histopathological screening did not reveal any organ‐specific accumulation or inflammation. In line with other investigations, the biodistribution of rGO remnants after macrophage uptake and potential adverse effects on organ functionality should be examined after several months of implantation.^[^ ^20^, ^57^ ^]^ Nevertheless, these results indicate that rGO remains in the body for a sufficient amount of time to allow tissue regeneration to occur.

### 2.4 Application of COL Induces Orthotopic Bone Formation

Following SCI, attachment of reactive astrocytes to collagen type I through an integrin‐N‐cadherin axis accelerated a phenotypic change into scar‐forming astrocytes.^[^ ^40^ ^]^ Moreover, as opposed to graphene gels, implantation of pristine COL cryogels led to increased astrogliosis upon SCI.^[^ ^24^ ^]^ Since this sequential phenotypic change impaired axonal regeneration and consequently hampered functional recovery,^[^ ^40^ ^]^ scaffolds composed of COL were implanted to serve as a negative control. However, even in the absence of any cellular or biochemical cues, orthotopic bone formation was observed within the lesion site 8 weeks after COL implantation (Figure S3A, Supporting Information). Similar to collagen sponges or devitalized ECM scaffolds used before, bone formation was evidenced by the presence of trabecular bone struts, bone marrow foci, and blood vessel infiltration.^[^ ^58^, ^59^ ^]^ In fact, COL potentially served as a front for new bone formation,^[^ ^60^ ^]^ recapitulating signs of material‐induced ossification.^[^ ^61^ ^]^ Orthotopic bone formation was observed in almost all animals (*n* = 5/7) that received a COL implant for 8 weeks (Figure S3B, Supporting Information). As evidenced by the assessment of consecutive tissue sections, bone growth was initiated at the site of the laminectomy and subsequently propagated ventrally into the lesion site. Notably, the immunological response to biomaterials seemed pivotal in this process, as orthotopic bone formation was absent in adECM implants of porcine origin compared to rat‐derived COL implants. Such orthotopic bone formation into the lesion site following SCI using COL‐based biomaterials has not yet been reported. These results urge caution when using COL‐based implants for SCI repair. In conclusion, this phenomenon renders the COL treatment group incomparable to the other treatment groups and was therefore excluded from further analysis.

### 2.5 Myelinated Axons in Host‐Material Interface and Periphery of adECM‐rGO Scaffolds

**Figure 3:**
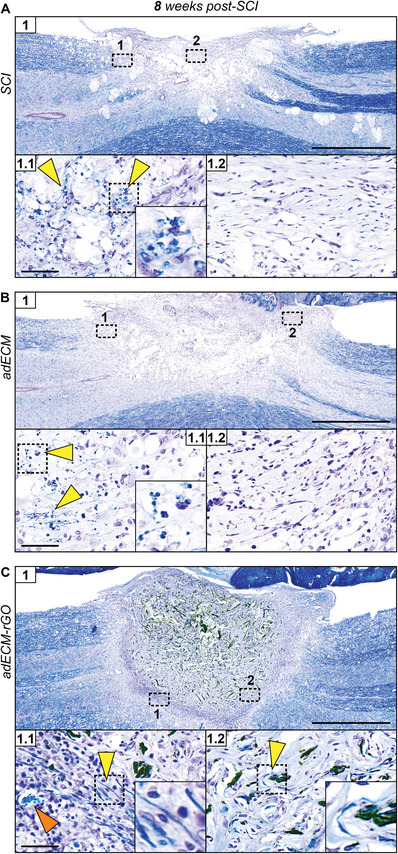
Presence of myelinated axons in the periphery of adECM‐rGO scaffold. Klüver‐Barrera histological staining was performed to visualize neuronal cell bodies (purple) and myelin (dark blue) in spinal cord tissue of the SCI (A), adECM (B), and adECM‐rGO (C) groups (from top to bottom) at 8 weeks post‐SCI. For each condition, two inserts have been included that contain higher magnification images of the peripheral (1) or more centrally located (2) regions of the lesion site. Yellow arrows represent myelin, and orange arrows depict blood vessels. Note that for SCI and adECM, myelin is often observed in an interrupted, degenerated morphology, in (A,B).1, and was not observed in central regions of the lesion sites. A strong presence of myelin was observed in the cell‐dense host‐material interface after adECM‐rGO implantation (C1.1), and these fiber‐like structural features were also present within the periphery of the scaffold (C1.2). Here, the additional insert shows that myelinated axons were in close contact with rGO remnants. Scale bars represent 1000 µm (overviews), or 50 µm (inserts).

The presence of neuronal cell bodies and myelinated axons following implantation was examined with a conventional Klüver–Barrera staining, of which the results are presented in **Figure** **3**. Upon SCI, axonal degeneration is characterized by axons forming enlarged, swollen endings that frequently group as retraction bulbs, chronically persistent at the injury margin.^[^ ^62^ ^]^ Regarding both the injury control (Figure 3A1.1) and adECM (Figure 3B1.1) implantation, the myelin observed in peripheral regions surrounding the lesion recapitulated many of the characteristics of such retraction bulbs. Moreover, myelin could not be observed within the lesion site of both groups (Figures 3A1.2, B1.2). Interestingly, myelinated axons were readily observed in the host‐material interface, directly adjacent to the adECM‐rGO scaffold (Figure 3C1.1). The highly vascularized, yet cellularly dense, interface allowed for substantial growth of myelinated axons along the border of the implant. More importantly, even though the presence of collagen in adECM remnants or matrix produced *de novo* is known to generate artifacts and thus false positives,^[^ ^63^ ^]^ the presence of myelinated axons was confirmed even beyond the host‐material interface (Figure 3C1.2). The typical, fiber‐like structural features, which are distinct from any artifacts, indicate that endogenous axons were capable of crossing the host‐material interface and subsequently infiltrating the adECM‐rGO implant. Moreover, myelinated axons were present directly adjacent to rGO remnants, in accordance with myelination being previously reported for GBMs.^[^ ^19^, ^24^ ^]^ These encouraging findings warrant further assessments to visualize axons sprouting into the GBM.

### 2.6 Increase of Axonal Growth only Observed in the Host‐Material Interface

**Figure 4:**
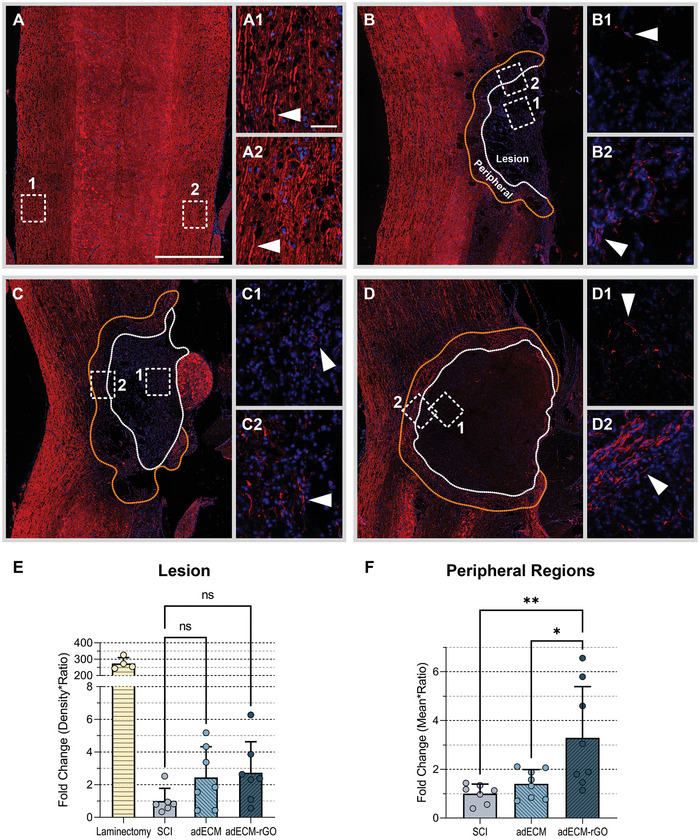
Increase of βIII‐Tubulin only in peripheral regions of the adECM‐rGO scaffold. Immunofluorescence staining against βIII‐Tubulin was performed to visualize axons in the healthy control (A) and axonal regeneration in the SCI (B), adECM (C), and adECM‐rGO (D) groups at 8 weeks post‐SCI. For each condition, two inserts have been included that contain higher magnification images of the peripheral (1) or more centrally located (2) regions of the lesion site. For each animal, a representative coronal section was selected and subsequently examined one time. Each data point refers to one animal and example images are representative of that specific group. White arrows depict the presence of βIII‐Tubulin. Normalized density of βIII‐Tubulin was quantified in the lesion (circumvented by a white dotted line) in panel E, as well as a 200 µm‐wide peripheral region surrounding the lesion site (circumvented by an orange dotted line) in panel F. Note that axonal sprouting was observed in both the adECM and adECM‐rGO scaffolds, although statistically significant alterations were not detected. However, in the cell‐dense perilesional border, adECM‐rGO scaffolds demonstrated a significantly increased presence of βIII‐Tubulin. Scale bars represent 1000 µm (overviews), or 20 µm (inserts).

Immunofluorescence staining against βIII‐tubulin was carried out to provide a conclusive answer to whether axonal regeneration is promoted following implantation of adECM‐rGO (**Figure** **4**). Microtubuli, such as βIII‐tubulin, play a critical role in axonal guidance and neurogenesis and βIII‐tubulin is considered one of the earliest cytoskeletal markers for neurons.^[^ ^64^ ^]^ Accordingly, the recent application of similar graphene‐based foams demonstrated a predominant expression of βIII‐tubulin over others in neurites,^[^ ^19^ ^]^ making this a suitable neuronal marker for this purpose. Figure 4 shows the expression of βIII‐tubulin in the healthy control (Figure 4A), injury control (Figure 4B), as well as the adECM (Figure 4C) and adECM‐rGO (Figure 4D) treatment groups. In line with the Klüver‐Barrera histological staining, the sprouting of axons into the adECM‐rGO scaffold was observed (Figure 4D1). However, a significantly increased intralesional presence of βIII‐tubulin could not be detected (Figure 4E). Moreover, the levels of βIII‐tubulin expression within these implant groups are far from those observed in the healthy control group and far from promising state‐of‐the‐art applications of conductive hydrogels following transection SCI.^[^ ^65^ ^]^ Nevertheless, since only bare adECM‐rGO scaffolds were implanted, lacking cellular or biochemical cues that are often present in state‐of‐the‐art applications, it is noteworthy that peripheral regions showed a significantly increased expression of βIII‐tubulin (Figure 4F). Confirming the Klüver‐Barrera staining results, axonal growth was observed predominantly within the host‐material interface. Moreover, axonal presence was observed even in regions that were expected to be heavily populated by scarring astrocytes, which will be discussed below. Therefore, it is plausible that, instead of the scarring process, the scaffold properties itself did not allow for sufficient scaffold penetration by βIII‐tubulin‐positive axons.

### 2.7 Implantation of adECM or adECM‐rGO did not affect Astrocyte Reactivity or Migration

Astrogliosis following SCI is hallmarked by reactive, hypertrophic astrocytes that migrate toward the site of injury and combine with CPSGs to form the glial scar.^[^ ^4^ ^]^ Although axonal growth is restricted by the scar and the cavity it encloses, the scarring process is essential to restrict inflammation and limit further injury progression.^[^ ^5^, ^6^ ^]^ Nevertheless, at chronic stages of SCI, this tightly interwoven meshwork of reactive astrocytes and CSPGs is almost impenetrable for any regenerating endogenous axon.^[^ ^66^ ^]^ Biomaterial therapies should not only limit astrogliosis but also provide structural support to drive directional endogenous glial migration, which may potentially inhibit scar formation at the host‐implant interface and foster neuronal regeneration.

**Figure 5:**
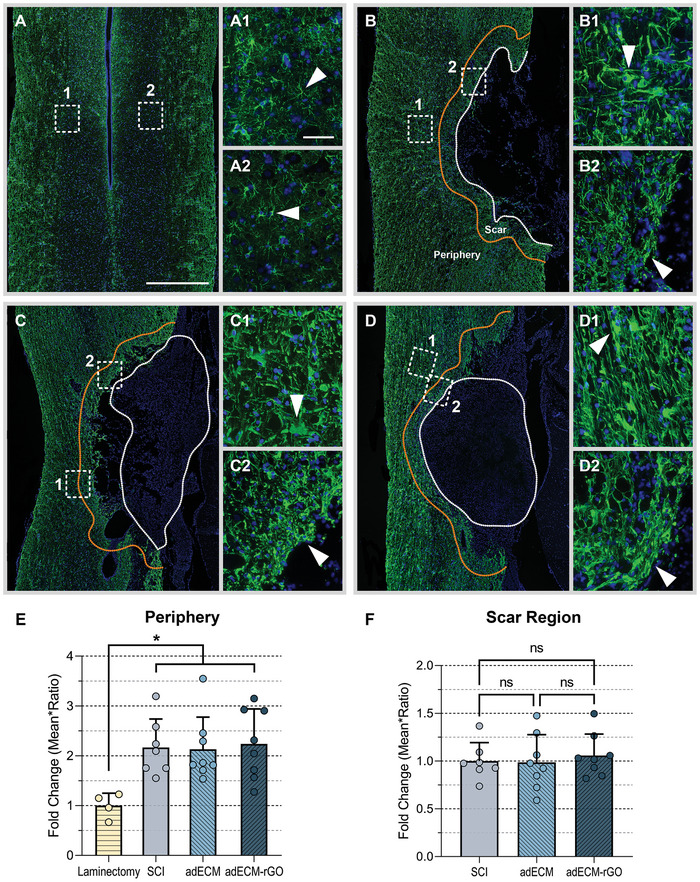
No attenuation of GFAP reactivity following adECM or adECM‐rGO implantation. Immunofluorescence staining against GFAP was performed to visualize astrocytes in the healthy control (A) and assess astrocyte reactivity and glial scar formation in the SCI (B), adECM (C), and adECM‐rGO (D) groups at 8 weeks post‐SCI. For each condition, two inserts have been included that contain higher magnification images of the peripheral (1) or scar (2) regions of the lesion site. For each animal, a representative coronal section was selected and subsequently examined one time. Each data point refers to one animal and example images are representative of that specific group. White arrows depict the presence of astrocytes, and the lesion site of scaffolds is circumvented by a white dotted line. Normalized density of GFAP was quantified in the spinal cord periphery in panel E, as well as a 200 µm‐wide scar region surrounding the lesion site (circumvented by an orange dotted line) in panel F. Note that GFAP density doubled following SCI, but no changes in glial scar formation were observed following adECM or adECM‐rGO implantation. Scale bars represent 1000 µm (overviews), or 20 µm (inserts).

After 8 weeks of implantation, a dense, cellular barrier surrounding the adECM‐rGO implant (Figure 1 C8) suggested ongoing astrogliosis, which is disputed by the fact that myelinated and βIII‐tubulin‐positive axons were strongly present within the host‐material interface. More insight into the astroglial response is shown in **Figure** **5**, which presents the expression of GFAP in the healthy control (Figure 5A), injury control (Figure 5B), adECM (Figure 5C), and adECM‐rGO (Figure 5D) treatment groups. Compared to astrocytes resident in healthy spinal cord tissue (Figure 5A1‐2), SCI caused astrocytes to undergo hypertrophy and process extension (Figures 5B–D1). This was confirmed by the two‐fold increased expression of GFAP (Figure 5E), a value that is regularly reported following the induction of SCI.^[^ ^67^ ^]^ Subsequent glial scarring was observed in all treatment groups and remained unaffected by treatment with either adECM or adECM‐rGO (Figure 5F). These findings corroborate the majority of existing reports describing an extremely limited effect of GBMs on astrocyte reactivity following SCI,^[^ ^17^, ^19^, ^20^, ^21^ ^]^ although some only with the incorporation of anti‐inflammatory drugs.^[^ ^68^ ^]^ Nevertheless, these results indicate that both adECM and adECM‐rGO were mechanically compliant with the spinal cord, as a mismatch in mechanical properties would trigger highly mechanosensitive astrocytes to undergo reactive gliosis.^[^ ^69^ ^]^ Similar to the aforementioned alternative GBMs, astrocytes failed to penetrate the scaffold and were restricted to the host‐material interface. During development, astrocytes serve as substrates supporting neuronal migration, as immature neurons physically attach to and migrate along astrocytic processes.^[^ ^66^ ^]^ Scaffold penetration by astrocytes, provided that these cells have neuroprotective features,^[^ ^70^ ^]^ could thus promote axonal pathfinding and neuronal regeneration. Integration of astrocytic processes was directly associated with axon regeneration into fluid‐like Schwann cell (SC) bridges, which correlated with improved functional recovery.^[^ ^71^ ^]^ Contrasting these results, a very recent application of graphene‐based cryogels led to attenuated scar formation, which was associated with decreased STAT3 signaling, and improved scaffold penetration.^[^ ^24^ ^]^ However, the authors did not evidence how GBMs directly affected STAT3 signaling and glial migration was not correlated with eventual axonal outgrowth.^[^ ^24^ ^]^ Since digestion of CSPG, as another example of scar attenuation, significantly promoted astrocyte integration and subsequent neurite outgrowth,^[^ ^72^ ^]^ future GBMs should pursue astrocyte penetration beyond the host‐material interface.

### 2.8 Increase of CSPG within adECM‐rGO Independent of Astrocyte Reactivity

**Figure 6:**
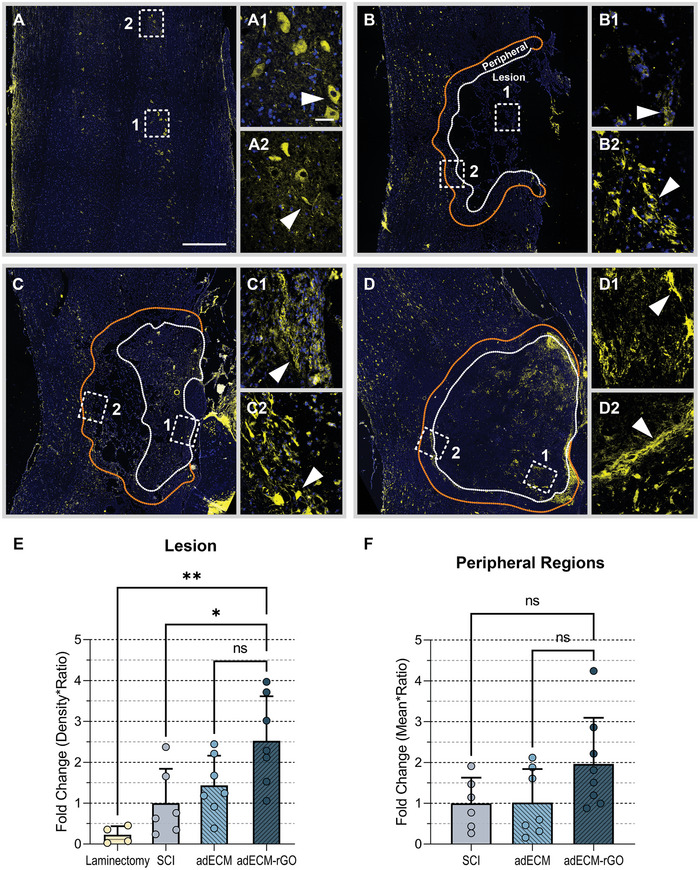
Increase of CSPG deposition within adECM‐rGO scaffold. Immunofluorescence staining against CSPG was performed to visualize proteoglycans, mostly inhibitory toward axonal regrowth, in the healthy control (A), SCI (B), adECM (C), and adECM‐rGO (D) groups at 8 weeks post‐SCI. For each condition, two inserts have been included that contain higher magnification images of the lesion (1) or peripheral scar (2) regions of the lesion site. For each animal, a representative coronal section was selected and subsequently examined one time. Each data point refers to one animal and example images are representative of that specific group. White arrows depict CSPG, and the lesion site of scaffolds is circumvented by a white dotted line. Normalized density of CSPG was quantified in the lesion in panel E, as well as a 200 µm‐wide peripheral region surrounding the lesion site (circumvented by an orange dotted line) in panel F. Note that CSPG expression significantly increased following adECM‐rGO implantation, but no changes were observed in the peripheral regions. Scale bars represent 1000 µm (overviews), or 20 µm (inserts).

Even though astrocyte reactivity was retained, a heterogeneous population of scar‐forming cells, such as microglia, fibroblasts, and pericytes, are well‐known contributors to the glial scar.^[^ ^3^ ^]^ Therefore, it was explored to what extent CSPG expression is affected by scaffold implantation. **Figure** **6** shows the expression of CSPG in the healthy control (Figure 6A), injury control (Figure 6B), adECM (Figure 6C), and adECM‐rGO (Figure 6D) treatment groups. Interestingly, CSPG expression was significantly increased within the adECM‐rGO scaffold, compared to the healthy and injury control (Figure 6E). Based on the fact that the majority of CSPGs are inhibitory toward axonal regeneration,^[^ ^73^ ^]^ this could partially explain the limited amount of axons capable of sprouting beyond the glial scar into the adECM‐rGO scaffolds. Features of CSPG‐rich scar formation were observed at the perilesional border, although only at occasional sites along the host‐material interface, and significant alterations were not observed (Figure 6F). Future endeavors should load GBMs, either with chondroitinase ABC (chABC)^[^ ^72^ ^]^ or GFs,^[^ ^65^ ^]^ to limit astrogliosis and generate permissive environments for nervous tissue repair. However, bringing (stem) cells into these materials is critical to the success of such loading regimens.^[^ ^74^ ^]^ The combination of cellular and biochemical cues present in scaffolds could potentially alleviate scar formation^[^ ^75^ ^]^ and instruct endogenous cells to more actively cross the host‐material interface.^[^ ^74^, ^76^, ^77^ ^]^ Moreover, the limited axonal penetration of the randomly oriented porous scaffolds observed here showed that axons will rarely extend beyond the lesion site without the proper guidance in terms of directionality.^[^ ^77^ ^]^

### 2.9 Improved Functional Recovery observed in adECM and adECM‐rGO‐Treated Animals

**Figure 7:**
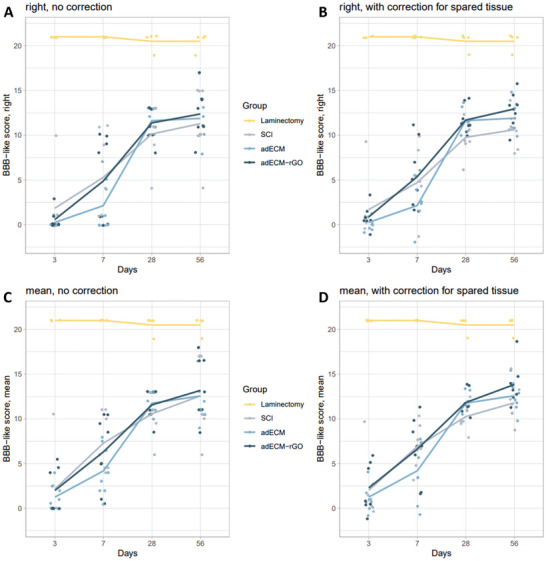
Functional recovery was not significantly improved following adECM and adECM‐rGO implantation. To analyze functional recovery, animals were scored according to the BBB locomotor rating scale, where 0 depicts no functionality and 21 depicts normal functional behavior, at 3 days post‐SCI and 1, 4, and 8 weeks post‐implantation. Recovery of the BBB‐like score for the right hindlimb before and after including “Spared Tissue” is presented in Panels A and B, respectively. Note that including “Spared Tissue” in the model led to a smaller dispersion of data points and a shift in the means of all groups, but no significant difference between the treatment groups was detected. Recovery of the BBB‐like score for both hindlimbs (mean) before and after including “Spared Tissue” in Panel C and D, respectively. The number of animals examined for each group throughout the experimental period: laminectomy (*n* = 4), SCI (*n* = 7), adECM (*n* = 8), adECM‐rGO (*n* = 8).

Functional recovery of the right hindlimb was measured according to a BBB‐like locomotor rating scale. Quantification of spared tissue revealed substantial variability in the extent of the lesion, ranging from incomplete to almost complete injuries (Figure S2B, Supporting Information). Given the fact that lesion size greatly affects functional recovery, similar to non‐human primates suffering from either incomplete or complete injuries,^[^ ^78^ ^]^ it is unsurprising this variability was represented in the BBB‐like score for locomotion recovery as well. More importantly, spared tissue was not normally distributed within each treatment group. To separate the effects of biomaterial treatment from the effects of the lesion^[^ ^73^ ^]^ and provide a more accurate assessment of functional recovery, statistical regression analysis included the quantified spared tissue as a correction to the original BBB‐like scores. For the sake of transparency, **Figure** **7** shows the effect of adECM and adECM‐rGO implantation on functional recovery, before (Figures 7A,C) and after (Figures 7B,D) correction for the right and mean BBB‐like scores, respectively. The correction to the BBB‐like score led to a smaller dispersion of data points regarding all treatment groups, but significant differences between treatment groups were not observed for both the right and mean BBB‐like scores.

Since the lesion size drastically affected functional recovery, a subgroup analysis was performed which only included animals that did not show any signs of recovery 1 week after implantation (Figure S4, Supporting Information). Within this subgroup, regression analysis (Table S1, Supporting Information) showed that implantation of adECM as well as adECM‐rGO significantly improved functional recovery regarding the right (Figures S4A,B, Supporting Information) and mean BBB‐like scores (Figures S4C,D, Supporting Information). By taking into account the distribution of the lesion size, as well as solely focusing on animals that did not show any signs of early recovery, the benefit of either treatment becomes evident. Such analyses could be applied in similar research in which the variability in SCI lesion size greatly hampers the functional outcome assessment. Summarizing, only very limited functional recovery was observed following implantation of adECM and adECM‐rGO, which can be partially attributed to the limited axonal sprouting and the significantly increased expression of CSPGs within the scaffold for the adECM‐rGO group. However, the substantial variability in lesion size, and consequently functional recovery, in the injury control greatly limited the subsequent comparison between treatment groups. The average BBB‐like score of animals that received an adECM‐rGO implant was ≈12.4, approaching the recovery observed following the application of state‐of‐the‐art conductive polymer hydrogels in similar SCI models.^[^ ^79^ ^]^ To have a meaningful impact on functional recovery, future rGO‐based composites should include various additional cues. For instance, introducing topographical guidance or exploiting various external stimuli in vitro previously led to enhanced neurogenesis on graphene‐ or rGO‐derived scaffolds.^[^ ^80^, ^81^, ^82^, ^83^ ^]^ Moreover, applying either electrical or chemical stimuli can accelerate the differentiation of stem cells into neurons as well as enhance functional neural network formation.^[^ ^84^, ^85^ ^]^ By combining several of these additional cues, future rGO‐based composites are expected to serve as effective neural interfaces for regenerative medicine.

## 3 Conclusion

To successfully repair the injured spinal cord and maximize functional recovery, an rGO‐based nanocomposite, supported by naturally derived adipose decellularized ECM, was evaluated for its effect on axonal regeneration, astrogliosis, and functional recovery following a right‐sided hemisection at T10. Whereas the COL control group showed detrimental orthotopic bone formation in the lesion site, the pristine adECM scaffold demonstrated a tissue‐restorative and angiogenic response. The adECM‐rGO nanocomposites completely restored the integrity of the spinal cord, and pivotal phagocytosis of rGO indicated ongoing clearance of the material. Although astrocyte reactivity remained unaltered, the increased production of CSPGs within the rGO‐based nanocomposites could partly explain the limited axonal ingrowth observed for these scaffolds. Nevertheless, an increased presence of βIII‐tubulin‐positive axons surrounded the adECM‐rGO nanocomposites. Even in the absence of any cellular or biochemical cues, implantation of both adECM and adECM‐rGO led to functional recovery, although no significant differences were detected due to the variability in lesion size affecting the reliability of the SCI model. Altogether, compared to pristine adECM, rGO‐based nanocomposites evoked a tissue‐regenerative response demonstrating the potential of these materials in repairing the injured spinal cord. Nevertheless, additional topographical and biochemical cues, along with additional stimuli including, e.g., stem cell transplantation and electrical stimulation, are still required to achieve meaningful recovery in future combinatorial therapies.

## 4 Experimental Section

### Fabrication of COL, adECM, and adECM‐rGO Scaffolds

Rat tail COL was commercially obtained (Merck, Darmstadt, Germany), and porcine adECM (Tecnalia, San Sebastián, Spain) was obtained similarly as reported before.^[^ ^33^ ^]^ Scaffolds of COL, adECM, and adECM‐rGO (50/50 wt%) were synthesized by solid‐liquid phase separation as previously described.^[^ ^38^ ^]^ A commercial dispersion of GO nanosheets (0.4 wt%) in water (Graphenea, San Sebastián, Spain) was first dialyzed against distilled water for 7 days to remove chemical impurities and subsequently freeze‐dried to avoid nanosheet agglomeration. The reduction of GO was thereafter performed by thermal annealing at 200 °C for 30 min.

COL or adECM was added to and dissolved in 0.1 m acetic acid for 48 h at a concentration of 10 mg mL^−1^. To generate adECM‐rGO scaffolds, rGO was first thoroughly dispersed in 0.1 m acetic acid by sonication. The adECM was then added and dissolved for 48 h at a concentration of 10 mg mL^−1^. Crosslinking was induced by adding the following coupling agents: 1‐Ethyl‐3‐(3‐dimethylaminopropyl)carbodiimide) (EDC) and N‐hydroxysuccinimide (NHS) at a concentration of EDC of 3.3 µmol mg^−1^ of adECM and an EDC:NHS molar ratio of 1. After 2 h, the solutions were cast on a 48‐well plate using 450 µL of solution per well, frozen at −20 °C overnight, and freeze‐dried for 2 days. Following freeze‐drying, scaffolds were thoroughly washed with distilled water to remove any residual reagents and subproducts, and freeze‐dried again. Extensive characterization of scaffolds can be found elsewhere.^[^ ^38^, ^39^ ^]^

### Animals

Adult female Sprague–Dawley rats (*n* = 50) of 289 ± 25.1 g. (Charles River; ‘s‐Hertogenbosch, The Netherlands) were used. All procedures were executed according to the regulations for animal experimentation in the Dutch Experiments on Animals Act (Wod) and the European Union (directive 2010/63/EU) and were approved by the Dutch Central Authority for Scientific Procedures on Animals (CCD; AVD10300202114976). Experimental details were described according to the ARRIVE guidelines.^[^ ^86^ ^]^ Rats were randomly divided into the following treatment groups: sham without injury (Laminectomy, *n* = 4), SCI‐only (SCI, *n* = 10), SCI with COL implantation (COL, *n* = 10), SCI with adECM implantation (adECM, *n* = 11), and SCI with adECM‐rGO implantation (adECM‐rGO, *n* = 11). Throughout the experiment, three animals (all adECM‐rGO) failed to recover urinary function and were prematurely excluded, according to exclusion criteria set a priori. One animal died intra‐operatively due to isoflurane‐induced respiratory depression.

Animals were allocated to treatment groups according to a randomized block design, where each block corresponded to a week of surgery. Randomization was performed by an independent researcher, meaning surgeons were blinded to group allocation. Except for the healthy control, experimental groups consisted of *n* = 3 animals to evaluate potential spinal cord regeneration 1, 3, and 5 weeks post‐SCI. Rats were housed in groups of 2 to 3 animals at a 12 h reversed light/dark cycle with food and water ad libitum. Since the mobility of the animal was heavily affected upon injury, moistened food was provided on the bottom of the cage during the first 7 days. Drinking water was more easily accessible due to the use of extended spouts. Animal cages were placed in the same room, yet the exact position was regularly changed, and behavioral assessments were performed at one specific time during the day, to minimize potential confounders.

### Surgical and Post‐Operative Procedures

Carprofen (3.33 mg kg^−1^; Bela‐Pharm, Vechta, Germany) was subcutaneously injected pre‐operatively, and post‐operatively for two additional days. Surgical procedures were performed under inhalation anesthesia with isoflurane (3–5% induction; 1.5–2.5% maintenance in 1:2 O_2_/air). Body temperature, heart rate, and respiratory frequency were consistently monitored during the surgical procedures. A mixture of bupivacaine (1.67 mg kg^−1^; Aurobindo, Baarn, The Netherlands) and lidocaine (3.33 mg kg^−1^; Fresenius Kabi, Bad Homburg, Germany) in 0.9% sterile NaCl was subcutaneously injected to provide local analgesia before a midline skin incision (≈2–3 cm). Fat and muscle tissue were carefully dissected along the midline to expose the T8–T11 vertebrae. After the removal of the T10 spinous process and lamina, a right‐sided hemisection (8 mm^3^) was created using iridectomy scissors. Bleeding was contained using Spongostan (Ethicon, Raritan, NJ, USA). Two independent surgeons generated the hemisection, blinded to treatment conditions. Upon completion of the injury, the treatment group of that specific animal was revealed. Scaffolds were resized to appropriate dimensions and implanted within the lesion site. In the case of the sham group, animals were only subjected to a laminectomy. The injury control group did receive an injury, but no scaffold was implanted. Buprenorphine (0.03 mg kg^−1^; Richter Pharma, Wels, Austria) was subcutaneously injected 15 min before waking up. Then, deep and superficial muscles were approximated using 4‐0 synthetic absorbable sutures (Ethicon), followed by skin closure (EZ Clips; Stoelting, Dublin, Ireland). During the first 7 postoperative days, animal welfare, with urinary function in particular, was extensively monitored.

### Tissue Processing and Histological Procedures

Following intermediate (1, 3, and 5 weeks post‐SCI) or final experimental time points (8 weeks post‐SCI), animals were sacrificed by carbon dioxide (CO_2_) inhalation. The spinal column region corresponding to T8–T12 was excised and fixed in 10% neutral buffered formalin for 24h. Tissue samples were then cleared from vertebrae as much as possible and subsequently decalcified in 10% EDTA for 5 days. Tissue samples were dehydrated through a graded series of ethanol, cleared with xylene, and finally embedded in paraffin. The entire spinal cord tissue was subsequently sectioned (5 µm thickness) along the longitudinal axis, from bottom to top, using a microtome.

### Histological Procedures and Analysis

To assess host‐biomaterial interaction, sections were used for conventional hematoxylin and eosin (H&E) staining. In addition, a Klüver–Barrera (KB) staining was performed to visualize the structural features of neurons and myelin sheaths. Following both histological procedures, samples were dehydrated through a graded series of ethanol, mounted, and subsequently scanned using the PANNORAMIC 1000 slide digitalization system (3DHISTECH, Budapest, Hungary). Automatically scanned slides were evaluated in CaseViewer (3DHISTECH). Spared spinal cord tissue, contralateral to the lesion, was quantified in CaseViewer. In brief, the thickness of spared tissue was measured at rostral, medial, and caudal sites along the lesion. This was repeated for *n* = 6 coronal sections per animal, and the smallest thickness of spared tissue in each section was then selected. Spared tissue for each animal was then calculated as the average of *n* = 6 sections. The lesion cavity was quantified using ImageJ software (NIH).

### Fluorescent Immunohistochemistry Procedures and Analysis

Spinal cord samples were examined for the presence of 1) βIII‐tubulin/TUJ‐1 for neurons, 2) glial fibrillary acidic protein (GFAP) for astrocytes, and 3) chondroitin sulfate proteoglycans (CSPG). Samples were processed similarly as described earlier.^[^ ^87^ ^]^ In brief, sections were heated in citrate buffer (10 mm; pH 5.85; Merck) for 10 min, followed by 5 min of trypsin incubation (0.075%) at 37 °C. Samples were then incubated with either mouse anti‐βIII‐tubulin (1:200; sc‐80005, Santa Cruz, Dallas, TX, USA), mouse anti‐GFAP (1:400; sc‐33673, Santa Cruz), or mouse anti‐CSPG (1:800; C8035, Merck), and subsequently with secondary antibodies conjugated to Alexa fluorescent probes. All samples were counterstained using 4′,6‐diamidino‐2‐phenylindole (DAPI), mounted, and imaged with an AxioImager Z2 (Zeiss, Oberkochen, Germany). The expression of βIII‐tubulin, GFAP, and CSPG was quantified in ImageJ software. The fluorescence intensity and area of βIII‐tubulin and CSPG were quantified both within the lesion site (‘Lesion’) and in a 200 µm‐wide perilesional border (“Perilesional”). The fluorescence intensity and area of GFAP were quantified in a 200 µm‐wide perilesional scar (“Scar Region”) and spinal cord tissue outside that perilesional scar (“Peripheral”). In each graph, “Mean” refers to the fluorescence intensity (staining – background) while “Ratio” refers to the measured area of positive staining, divided by the area of lesion/implant or periphery in which the positive staining was quantified. All results are presented as fold change in expression, compared to the SCI‐only control group.

### Functional Recovery Assessment

Hindlimb locomotor function recovery, including limb movement, paw placement, coordination, and gait, was evaluated using the Basso, Beattie, and Bresnahan (BBB) rating scale^[^ ^88^ ^]^ before surgery, and 3, 7, 28, and 56 days post‐SCI. Animals were placed in an open field and locomotor behavior was filmed for at least 4 min. An independent examiner, blinded to the treatment group, evaluated these videos, and recovery of locomotor function was presented as a BBB‐like score. The experimental model involved a right‐sided injury, but the BBB‐like score represents the recovery of locomotor functionality of both hindlimbs (BBB‐like score mean) or solely the right hindlimb (BBB‐like score right).

### Statistical Analysis

All data were expressed as mean ± standard deviation. For quantification of lesion cavity, spared tissue, and immunofluorescence, statistical analysis was performed using one‐way ANOVA with a Bonferroni's post‐hoc test in Prism (GraphPad, San Diego, CA). Regarding the behavioral assessment, two linear regression models were used to analyze the BBB‐like scores over time. As in each rat, multiple measurements can be evaluated (one per point in time), and a multilevel model was used with a random intercept for each rat. The dependent variable was either the BBB‐like score of both hindlimbs (mean) or the BBB‐like score of the right hindlimb (right). Independent variables were the group assignment (laminectomy, SCI, adECM, and adECM‐rGO), and point in time (3, 7, 28, and 56 days post‐SCI), both as categorical variables. As an interaction between group and time was potentially available, this was added to the models as well. To elucidate the role of spared tissue in the development of the mean and right BBB‐like scores over time, an additional model was analyzed as an extension of the models described, by adding the amount of spared tissue as a continuous variable in interaction with time to the regression model. The regression analyses were performed with R version 4.4.0. For all statistical assessments, a *p*‐value < 0.05 was considered statistically significant.

## Conflict of Interest

The authors declare no conflict of interest.

## Author Contributions

K.V. did conceptualization, methodology, validation, formal analysis, investigation, data curation, wrote the original draft, did review and editing, visualization, project administration. L.B. performed formal analysis and wrote the original draft. N.B. did the investigation. E.M.B. did software, formal analysis, and wrote the original draft. S.C.‐D. did formal analysis and supervision. S.C.G.L. did conceptualization, and wrote the review and editing, supervision. P.A.A.P.M. did the conceptualization, supervision, and funding acquisition. A.K. did conceptualization, formal analysis, investigation, and wrote the original draft, did review & editing, supervision, project administration. X.F.W. did conceptualization, resources, and wrote the original draft, did review & editing, supervision, funding acquisition.

## Supporting information



Supporting Information

## Data Availability

The data that support the findings of this study are available from the corresponding author upon reasonable request.

## References

[1] V. Carrard , S. Kunz , C. Peter , Spinal Cord 2021, 59, 398. 33235298 10.1038/s41393-020-00582-5

[2] E. H. Roels , B. Aertgeerts , D. Ramaekers , K. Peers , Spinal Cord 2016, 54, 2. 26305872 10.1038/sc.2015.133

[3] A. Alizadeh , S. M. Dyck , S. Karimi‐Abdolrezaee , Front. Neurol. 2019, 10, 282. 30967837 10.3389/fneur.2019.00282 PMC6439316

[4] M. V. Sofroniew , Trends Neurosci. 2009, 32, 638. 19782411 10.1016/j.tins.2009.08.002 PMC2787735

[5] C. S. Ahuja , J. R. Wilson , S. Nori , M. R. N. Kotter , C. Druschel , A. Curt , M. G. Fehlings , Nat. Rev. Dis. Primers 2017, 3, 17018. 28447605 10.1038/nrdp.2017.18

[6] T. Führmann , P. N. Anandakumaran , M. S. Shoichet , Adv. Healthcare Mater. 2017, 6, 1601130. 10.1002/adhm.201601130 28247563

[7] G. Courtine , M. V. Sofroniew , Nat. Med. 2019, 25, 898. 31160817 10.1038/s41591-019-0475-6

[8] K. D. Kim , K. S. Lee , D. Coric , J. S. Harrop , N. Theodore , R. M. Toselli , Neurosurgery 2022, 90, 668. 35442254 10.1227/neu.0000000000001932 PMC9067089

[9] O. Akhavan , J. Mater. Chem. B 2016, 4, 3169. 32263253 10.1039/c6tb00152a

[10] A. F. Girão , M. C. Serrano , A. Completo , P. A. A. P. Marques , ACS Nano 2022, 16, 13430. 36000717 10.1021/acsnano.2c04756 PMC9776589

[11] T. H. Kim , S. Shah , L. Yang , P. T. Yin , M. K. Hossain , B. Conley , J. W. Choi , K. B. Lee , ACS Nano 2015, 9, 3780. 25840606 10.1021/nn5066028 PMC5808889

[12] J. Litowczenko , J. K. Wychowaniec , K. Załęski , Ł. Marczak , C. J. C. Edwards‐Gayle , K. Tadyszak , B. M. Maciejewska , Biomater. Adv. 2023, 154, 213653. 37862812 10.1016/j.bioadv.2023.213653

[13] O. Akhavan , E. Ghaderi , J. Mater. Chem. B 2013, 1, 6291. 32261702 10.1039/c3tb21085e

[14] O. Akhavan , E. Ghaderi , M. Shahsavar , Carbon 2013, 59, 200.

[15] M. Catanesi , G. Panella , E. Benedetti , G. Fioravanti , F. Perrozzi , L. Ottaviano , L. D. Leandro , M. Ardini , F. Giansanti , M. Angelo , V. Castelli , F. Angelucci , R. Ippoliti , A. Cimini , Nanomedicine 2018, 13, 3091. 30451074 10.2217/nnm-2018-0269

[16] E. López‐Dolado , A. González‐Mayorga , M. T. Portolés , M. J. Feito , M. L. Ferrer , F. Del Monte , M. C. Gutiérrez , M. C. Serrano , Adv. Healthcare Mater. 2015, 4, 1861. 10.1002/adhm.201500333 26115359

[17] A. H. Palejwala , J. S. Fridley , J. A. Mata , E. L. Samuel , T. G. Luerssen , L. Perlaky , T. A. Kent , J. M. Tour , A. Jea , Surg. Neurol. Int. 2016, 7, 75. 27625885 10.4103/2152-7806.188905 PMC5009578

[18] E. López‐Dolado , A. González‐Mayorga , M. C. Gutiérrez , M. C. Serrano , Biomaterials 2016, 99, 72. 27214651 10.1016/j.biomaterials.2016.05.012

[19] A. Domínguez‐Bajo , A. González‐Mayorga , C. R. Guerrero , F. J. Palomares , R. García , E. López‐Dolado , M. C. Serrano , Biomaterials 2019, 192, 461. 30502723 10.1016/j.biomaterials.2018.11.024

[20] A. Domínguez‐Bajo , A. González‐Mayorga , E. López‐Dolado , C. Munuera , M. García‐Hernández , M. C. Serrano , ACS Biomater. Sci. Eng. 2020, 6, 2401. 33455347 10.1021/acsbiomaterials.0c00345

[21] S. Pan , Z. Qi , Q. Li , Y. Ma , C. Fu , S. Zheng , W. Kong , Q. Liu , X. Yang , Artif. Cells, Nanomed. Biotechnol. 2019, 47, 651. 30829545 10.1080/21691401.2019.1575843

[22] B. Yang , P. B. Wang , N. Mu , K. Ma , S. Wang , C. Y. Yang , Z. B. Huang , Y. Lai , H. Feng , G. F. Yin , T. N. Chen , C. S. Hu , Neural Regener. Res. 2021, 16, 1829. 10.4103/1673-5374.306095 PMC8328790 33510090

[23] A. Yari‐Ilkhchi , A. Ebrahimi‐Kalan , M. Farhoudi , M. Mahkam , RSC Adv. 2021, 11, 19992. 35479903 10.1039/d1ra00861g PMC9033813

[24] G. Agarwal , A. Roy , H. Kumar , A. Srivastava , Biomater. Adv. 2022, 139, 212971. 35882128 10.1016/j.bioadv.2022.212971

[25] W. Jiang , X. Zhang , S. Yu , F. Yan , J. Chen , J. Liu , C. Dong , Exp. Neurol. 2023, 368, 114506. 37597763 10.1016/j.expneurol.2023.114506

[26] J. Y. Hong , Y. Seo , G. Davaa , H. W. Kim , S. H. Kim , J. K. Hyun , Acta Biomater. 2020, 101, 357. 31711898 10.1016/j.actbio.2019.11.012

[27] B. Q. Lai , Y. R. Bai , W. T. Han , B. Zhang , S. Liu , J. H. Sun , J. L. Liu , G. Li , X. Zeng , Y. Ding , Y. H. Ma , L. Zhang , Z. H. Chen , J. Wang , Y. Xiong , J. H. Wu , Q. Quan , L. Y. Xing , H. B. Zhang , Y. S. Zeng , Bioactive Mater. 2021, 11, 15. 10.1016/j.bioactmat.2021.10.005 PMC8661248 34938909

[28] D. Tukmachev , S. Forostyak , Z. Koci , K. Zaviskova , I. Vackova , K. Vyborny , I. Sandvig , A. Sandvig , C. J. Medberry , S. F. Badylak , E. Sykova , S. Kubinova , Tissue Eng., Part A 2016, 22, 306. 26729284 10.1089/ten.tea.2015.0422 PMC4799710

[29] Z. Kočí , K. Výborný , J. Dubišová , I. Vacková , A. Jäger , O. Lunov , K. Jiráková , Š. Kubinová , Tissue Eng. Part C, Methods 2017, 23, 333. 28471271 10.1089/ten.TEC.2017.0089

[30] Z. Wang , R. Liu , Y. Liu , Y. Zhao , Y. Wang , B. Lu , H. Li , C. Ju , W. Wu , X. Gao , H. Xu , S. Cheng , Y. Cao , S. Jia , C. Hu , L. Zhu , D. Hao , ACS Biomater. Sci. Eng. 2024, 10, 3218. 38593429 10.1021/acsbiomaterials.4c00067

[31] J. S. Choi , Y. C. Choi , J. D. Kim , E. J. Kim , H. Y. Lee , I. C. Kwon , Y. W. Cho , Macromol. Res. 2014, 22, 932.

[32] J. H. Kim , C. H. Jo , H. R. Kim , Y. I. Hwang , Stem Cells Int. 2018, 8429042. 29760736 10.1155/2018/8429042 PMC5901833

[33] M. Cicuéndez , L. Casarrubios , M. J. Feito , I. Madarieta , N. Garcia‐Urkia , O. Murua , B. Olalde , N. Briz , R. Diez‐Orejas , M. T. Portolés , Int. J. Mol. Sci. 2021, 22, 3847. 33917732 10.3390/ijms22083847 PMC8068109

[34] Z. L. Chen , S. Strickland , J. Cell Biol. 2003, 163, 889. 14638863 10.1083/jcb.200307068 PMC2173689

[35] A. Lukomska , B. A. Rheaume , M. P. Frost , W. C. Theune , J. Xing , A. Damania , E. F. Trakhtenberg , Exp. Neurol. 2024, 379, 114877. 38944331 10.1016/j.expneurol.2024.114877 PMC11283980

[36] A. E. Haggerty , M. M. Marlow , M. Oudega , Neurosci. Lett. 2017, 652, 50. 27702629 10.1016/j.neulet.2016.09.053

[37] M. Cicuéndez , A. García‐Lizarribar , L. Casarrubios , M. J. Feito , F. J. Fernández‐San‐Argimiro , N. García‐Urkia , O. Murua , I. Madarieta , B. Olalde , R. Diez‐Orejas , M. T. Portolés , Biomater. Adv. 2024, 159, 213794. 38367317 10.1016/j.bioadv.2024.213794

[38] N. Barroca , D. M. da Silva , S. C. Pinto , J. P. M. Sousa , K. Verstappen , A. Klymov , F. J. Fernández‐San‐Argimiro , I. Madarieta , O. Murua , B. Olalde , L. Papadimitriou , K. Karali , K. Mylonaki , E. Stratakis , A. Ranella , P. A. A. P. Marques , Biomater. Adv. 2023, 148, 213351. 36842343 10.1016/j.bioadv.2023.213351

[39] K. Verstappen , A. Klymov , M. Cicuéndez , D. Silva , N. Barroca , F. J. Fernández‐San‐Argimiro , I. Madarieta , L. Casarrubios , M. J. Feito , R. Diez‐Orejas , R. Ferreira , S. C. G. Leeuwenburgh , M. T. Portolés , P. A. A. P. Marques , X. F. Walboomers , Mater. Today Bio. 2024, 26, 101059. 10.1016/j.mtbio.2024.101059 PMC11061343 38693996

[40] M. Hara , K. Kobayakawa , Y. Ohkawa , H. Kumamaru , K. Yokota , T. Saito , K. Kijima , S. Yoshizaki , K. Harimaya , Y. Nakashima , S. Okada , Nat. Med. 2017, 23, 818. 28628111 10.1038/nm.4354

[41] A. Polo‐Montalvo , M. Cicuéndez , L. Casarrubios , N. Barroca , D. da Silva , M. J. Feito , R. Diez‐Orejas , M. C. Serrano , P. A. A. P. Marques , M. T. Portolés , Nanoscale 2023, 15, 17173. 37853851 10.1039/d3nr03145d

[42] M. J. Feito , M. Cicuéndez , L. Casarrubios , R. Diez‐Orejas , S. Fateixa , D. Silva , N. Barroca , P. A. A. P. Marques , M. T. Portolés , Int. J. Mol. Sci. 2022, 23, 10625. 36142540 10.3390/ijms231810625 PMC9506555

[43] O. Akhavan , Carbon 2015, 81, 158.

[44] E. Lilley , M. R. Andrews , E. J. Bradbury , H. Elliott , P. Hawkins , R. M. Ichiyama , J. Keeley , A. T. Michael‐Titus , L. D. F. Moon , S. Pluchino , J. Riddell , K. Ryder , P. K. Yip , Exp. Neurol. 2020, 328, 113273. 32142803 10.1016/j.expneurol.2020.113273

[45] K. Verstappen , R. Aquarius , A. Klymov , K. E. Wever , L. Damveld , S. C. G. Leeuwenburgh , R. H. M. A. Bartels , C. R. Hooijmans , X. F. Walboomers , Tissue Eng. Part B, Rev 2022, 28, 1169. 34915758 10.1089/ten.teb.2021.0194 PMC9805871

[46] J. K. Hyun , H. W. Kim , J. Tissue Eng. 2010, 1, 650857. 10.4061/2010/650857 PMC3042682 21350645

[47] K. Zweckberger , C. S. Ahuja , Y. Liu , J. Wang , M. G. Fehlings , Acta Biomater. 2016, 42, 77. 27296842 10.1016/j.actbio.2016.06.016

[48] G. E. Rooney , T. Endo , S. Ameenuddin , B. Chen , S. Vaishya , L. Gross , T. K. Schiefer , B. L. Currier , R. J. Spinner , M. J. Yaszemski , A. J. Windebank , J. Neurosurg. Spine 2009, 11, 432. 19929340 10.3171/2009.4.SPINE08784 PMC2981802

[49] Z. You , X. Gao , X. Kang , W. Yang , T. Xiong , Y. Li , F. Wei , Y. Zhuang , T. Zhang , Y. Sun , H. Shen , J. Dai , Bioact. Mater. 2023, 29, 36. 37621772 10.1016/j.bioactmat.2023.06.019 PMC10444976

[50] V. Mastrullo , W. Cathery , E. Velliou , P. Madeddu , P. Campagnolo , Front. Bioeng. Biotechnol. 2020, 8, 188. 32266227 10.3389/fbioe.2020.00188 PMC7099606

[51] P. P. Partyka , Y. Jin , J. Bouyer , A. DaSilva , G. A. Godsey , R. G. Nagele , I. Fischer , P. A. Galie , Sci. Rep. 2019, 9, 2190. 30778117 10.1038/s41598-019-38558-y PMC6379421

[52] L. R. Nih , S. Gojgini , S. T. Carmichael , T. Segura , Nat. Mater. 2018, 17, 642. 29784996 10.1038/s41563-018-0083-8 PMC6019573

[53] H. Amani , E. Mostafavi , H. Arzaghi , S. Davaran , A. Akbarzadeh , O. Akhavan , H. Pazoki‐Toroudi , T. J. Webster , ACS Biomater. Sci. Eng. 2019, 5, 193. 33405863 10.1021/acsbiomaterials.8b00658

[54] C. M. Girish , A. Sasidharan , G. S. Gowd , S. Nair , M. Koyakutty , Adv. Healthcare Mater. 2013, 2, 1489. 10.1002/adhm.201200489 23554400

[55] L. Newman , D. A. Jasim , E. Prestat , N. Lozano , I. de Lazaro , Y. Nam , B. M. Assas , J. Pennock , S. J. Haigh , C. Bussy , K. Kostarelos , ACS Nano 2020, 14, 10168. 32658456 10.1021/acsnano.0c03438 PMC7458483

[56] V. C. Sanchez , A. Jachak , R. H. Hurt , A. B. Kane , Chem. Res. Toxicol. 2012, 25, 15. 21954945 10.1021/tx200339h PMC3259226

[57] D. Moura , S. Rohringer , H. P. Ferreira , A. T. Pereira , C. C. Barrias , F. D. Magalhães , H. Bergmeister , I. C. Gonçalves , Acta Biomater. 2024, 173, 351. 37984630 10.1016/j.actbio.2023.11.012

[58] H. Katagiri , Y. El Tawil , N. P. Lang , J. C. Imber , A. Sculean , M. Fujioka‐Kobayashi , N. Saulacic , Biomedicines 2021, 9, 143. 33540647 10.3390/biomedicines9020143 PMC7913003

[59] S. Pigeot , P. E. Bourgine , J. Claude , C. Scotti , A. Papadimitropoulos , A. Todorov , C. Epple , G. M. Peretti , I. Martin , Int. J. Mol. Sci. 2020, 21, 7233. 33008121 10.3390/ijms21197233 PMC7582540

[60] U. Kuchler , T. Rybaczek , T. Dobask , P. Heimel , S. Tangl , J. Klehm , M. Menzel , R. Gruber , Clin. Oral Implants Res. 2018, 29, 381. 29453780 10.1111/clr.13133

[61] D. Li , Y. Jiang , P. He , Y. Li , Y. Wu , W. Lei , N. Liu , J. D. de Bruijn , H. Zhang , H. Zhang , P. Ji , H. Yuan , M. Li , Adv. Sci. 2023, 10, e2207224. 10.1002/advs.202207224 PMC10214238 36970815

[62] C. E. Hill , Neurosci. Lett. 2017, 652, 11. 27825985 10.1016/j.neulet.2016.11.002

[63] H. Suzuki , T. Kanchiku , Y. Imajo , Y. Yoshida , N. Nishida , T. Gondo , S. Yoshii , T. Taguchi , Medical Mol. Morphol. 2015, 48, 214. 10.1007/s00795-015-0104-5 25982872

[64] A. M. P. Duly , F. C. L. Kao , W. S. Teo , M. Kavallaris , Front. Cell Dev. Biol. 2022, 10, 851542. 35573698 10.3389/fcell.2022.851542 PMC9096907

[65] C. Fan , W. Yang , L. Zhang , H. Cai , Y. Zhuang , Y. Chen , Y. Zhao , J. Dai , Biomaterials 2022, 288, 121689. 35931574 10.1016/j.biomaterials.2022.121689

[66] C. C. Winter , K. S. Katiyar , N. S. Hernandez , Y. J. Song , L. A. Struzyna , J. P. Harris , D. K. Cullen , Acta Biomater. 2016, 38, 44. 27090594 10.1016/j.actbio.2016.04.021 PMC5457669

[67] A. Karalija , L. N. Novikova , P. J. Kingham , M. Wiberg , L. N. Novikov , PLoS One 2012, 7, e41086. 22815926 10.1371/journal.pone.0041086 PMC3398872

[68] K. Zhang , J. Li , J. Jin , J. Dong , L. Li , B. Xue , W. Wang , Q. Jiang , Y.i Cao , Mater. Des. 2020, 196, 109092.

[69] P. Moshayedi , G. Ng , J. C. Kwok , G. S. Yeo , C. E. Bryant , J. W. Fawcett , K. Franze , J. Guck , Biomaterials 2014, 35, 3919. 24529901 10.1016/j.biomaterials.2014.01.038

[70] S. A. Liddelow , K. A. Guttenplan , L. E. Clarke , F. C. Bennett , C. J. Bohlen , L. Schirmer , M. L. Bennett , A. E. Münch , W. S. Chung , T. C. Peterson , D. K. Wilton , A. Frouin , B. A. Napier , N. Panicker , M. Kumar , M. S. Buckwalter , D. H. Rowitch , V. L. Dawson , T. M. Dawson , B. Stevens , B. A. Barres , Nature 2017, 541, 481. 28099414 10.1038/nature21029 PMC5404890

[71] R. R. Williams , M. Henao , D. D. Pearse , M. B. Bunge , Cell Transplant. 2015, 24, 115. 24152553 10.3727/096368913X674657 PMC4809058

[72] P. M. Warren , M. R. Andrews , M. Smith , K. Bartus , E. J. Bradbury , J. Verhaagen , J. W. Fawcett , J. C. F. Kwok , Sci. Rep. 2020, 10, 11262. 32647242 10.1038/s41598-020-67526-0 PMC7347606

[73] A. Raspa , L. Carminati , R. Pugliese , F. Fontana , F. Gelain , J. Control. Release 2021, 330, 1208. 33229053 10.1016/j.jconrel.2020.11.027

[74] D. Liu , G. Lu , B. Shi , H. Ni , J. Wang , Y. Qiu , L. Yang , Z. Zhu , X. Yi , X. Du , B. Shi , Adv. Healthcare Mater. 2023, 12, e2300123. 10.1002/adhm.202300123 36989238

[75] S. Song , Y. Li , J. Huang , S. Cheng , Z. Zhang , Biomater. Adv. 2023, 148, 213385. 36934714 10.1016/j.bioadv.2023.213385

[76] S. Yao , F. He , Z. Cao , Z. Sun , Y. Chen , H. Zhao , X. Yu , X. Wang , Y. Yang , F. Rosei , L. N. Wang , ACS Biomater. Sci. Eng. 2020, 6, 1165. 33464837 10.1021/acsbiomaterials.9b01557

[77] M. I. Günther , N. Weidner , R. Müller , A. Blesch , Acta Biomater. 2015, 27, 140. 26348141 10.1016/j.actbio.2015.09.001

[78] J. R. Slotkin , C. D. Pritchard , B. Luque , J. Ye , R. T. Layer , M. S. Lawrence , T. M. O'Shea , R. R. Roy , H. Zhong , I. Vollenweider , V. R. Edgerton , G. Courtine , E. J. Woodard , R. Langer , Biomaterials 2017, 123, 63. 28167393 10.1016/j.biomaterials.2017.01.024

[79] L. Zhou , L. Fan , X. Yi , Z. Zhou , C. Liu , R. Fu , C. Dai , Z. Wang , X. Chen , P. Yu , D. Chen , G. Tan , Q. Wang , C. Ning , ACS Nano 2018, 12, 10957. 30285411 10.1021/acsnano.8b04609

[80] Y. Wang , W. C. Lee , K. K. Manga , P. K. Ang , J. Lu , Y. P. Liu , C. T. Lim , K. P. Loh , Adv. Mater. 2012, 24, 4285. 22689093 10.1002/adma.201200846

[81] O. Akhavan , E. Ghaderi , Nanoscale 2013, 5, 10316. 24056702 10.1039/c3nr02161k

[82] O. Akhavan , E. Ghaderi , J. Mater. Chem. B 2014, 2, 5602. 32262194 10.1039/c4tb00668b

[83] O. Akhavan , E. Ghaderi , S. A. Shirazian , Colloids Surf. B Biointerfaces 2015, 126, 313. 25578421 10.1016/j.colsurfb.2014.12.027

[84] O. Akhavan , E. Ghaderi , S. A. Shirazian , R. Rahighi , Carbon 2016, 97, 71.

[85] M. Tang , Q. Song , N. Li , Z. Jiang , R. Huang , G. Cheng , Biomaterials 2013, 34, 6402. 23755830 10.1016/j.biomaterials.2013.05.024

[86] N. Percie du Sert , V. Hurst , A. Ahluwalia , S. Alam , M. T. Avey , M. Baker , W. J. Browne , A. Clark , I. C. Cuthill , U. Dirnagl , M. Emerson , P. Garner , S. T. Holgate , D. W. Howells , N. A. Karp , S. E. Lazic , K. Lidster , C. J. MacCallum , M. Macleod , E. J. Pearl , H. Würbel , PLoS biology 2020, 18, e3000410. 32663219 10.1371/journal.pbio.3000410 PMC7360023

[87] H. Tang , J. F. A. Husch , Y. Zhang , J. A. Jansen , F. Yang , J. J. J. P. van den Beucken , J. Tissue Eng. Regener. Med. 2019, 13, 785. 10.1002/term.2826 PMC6594112 30771241

[88] D. M. Basso , M. S. Beattie , J. C. Bresnahan , Journal of neurotrauma 1995, 12, 1. 7783230 10.1089/neu.1995.12.1

