# Supplementary material for: Application of Adipose Extracellular Matrix and Reduced Graphene Oxide Nanocomposites for Spinal Cord Injury Repair
Source: Adv Healthc Mater. 2024 Dec 12;14(3):2402775. doi: 10.1002/adhm.202402775 (PMC11773115; doi:10.1002/adhm.202402775)
Supplement: Supplementary file 1 — Supporting Information [file ADHM-14-0-s001.docx]

Supporting information for:

Application of Adipose Extracellular Matrix and Reduced Graphene Oxide Nanocomposites for Spinal Cord Injury Repair

**Authors:**

Kest Verstappen^1^, Lara Bieler^2,3^, Nathalie Barroca^4^, Ewald M. Bronkhorst^1^, Sebastien Couillard-Despres^2,3^, Sander C.G. Leeuwenburgh^1^, Paula A.A.P. Marques^4^, Alexey Klymov^1^, and X. Frank Walboomers^1^*

*Corresponding author: X. Frank Walboomers (Frank.Walboomers@radboudumc.nl)

**Affiliations:**

^1^Department of Dentistry-Regenerative Biomaterials, Radboud University Medical Center; 6525 EX, Nijmegen, The Netherlands.

^2^Institute of Experimental Neuroregeneration, Paracelsus Medical University; 5020, Salzburg, Austria.

^3^Austrian Cluster for Tissue Regeneration; 1200, Vienna, Austria.

^4^Centre for Mechanical Technology and Automation (TEMA), Intelligent Systems Associate Laboratory (LASI), Department of Mechanical Engineering, University of Aveiro; 3810-193, Aveiro, Portugal.


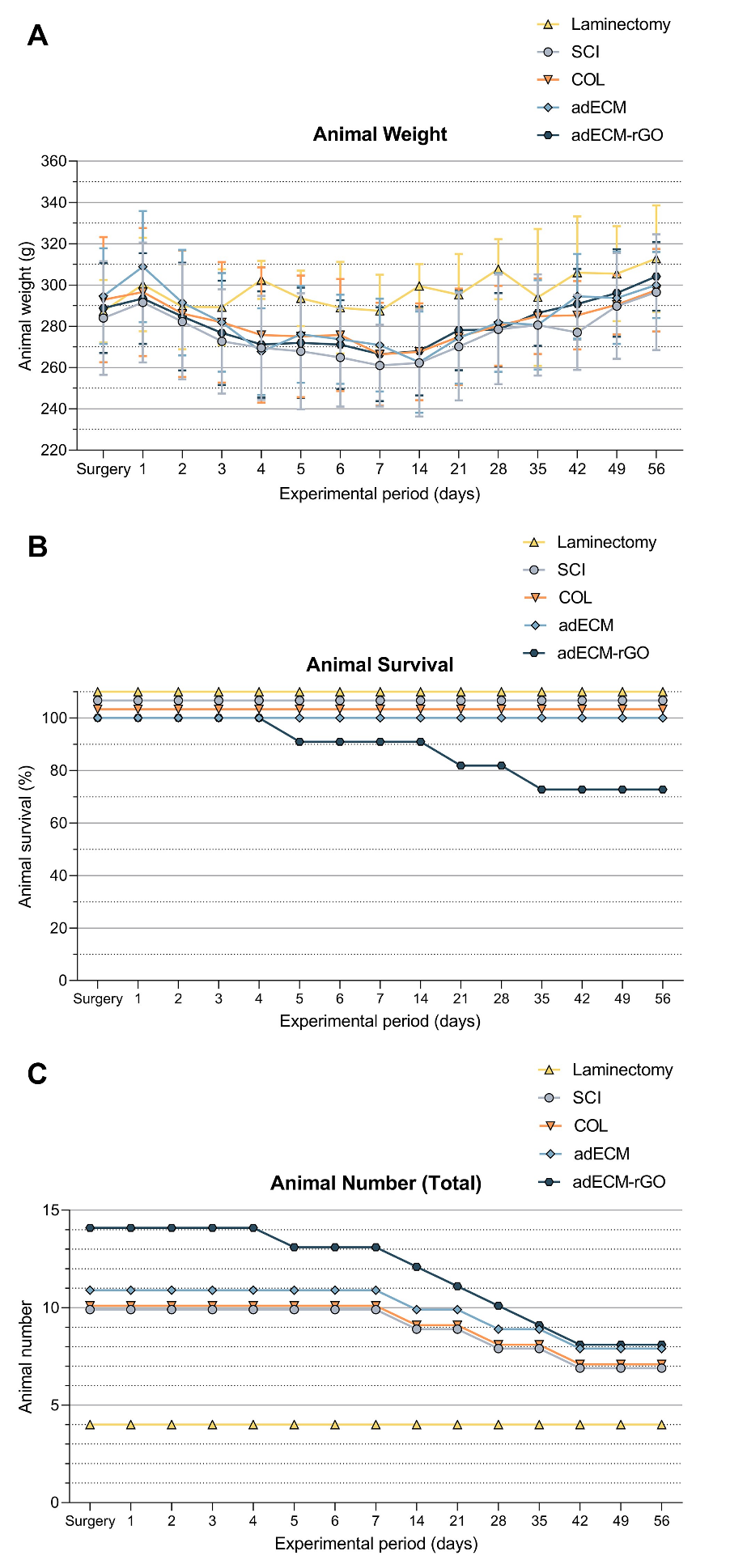
Supporting Figures

**Figure S1**. **Implantation of COL, adECM, and adECM-rGO did not affect animal welfare.**

Distribution of animal weight (A), animal survival (B), and animal number (C) are presented throughout the experimental period of 8 weeks. Note that weight did not differ between treatment groups. Notably, only animals from the adECM-rGO group were prematurely excluded due to urinary problems. For the sake of transparency, animal numbers are presented, demonstrating when animal were excluded, either due to urinary problems or due to pre-established points of sacrifice (1, 3, and 5 weeks post-SCI).

**
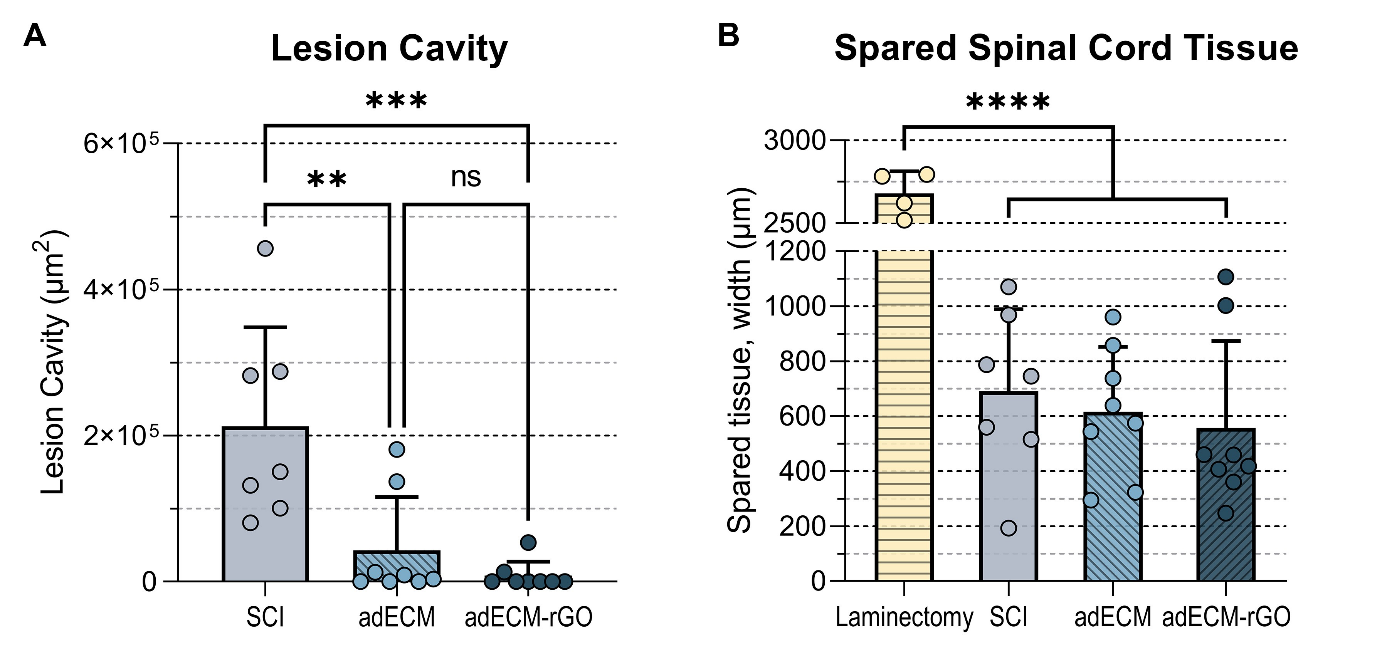
**

**Figure S2**. **Significant restoration of tissue architecture by scaffold implantation.** Presented are quantifications of lesion cavities and spared spinal cord tissue at 8 weeks post-SCI. Note that lesion cavities were significantly reduced following the implantation of adECM or adECM-rGO scaffolds. However, quantification of spared spinal cord tissue demonstrated that there were large variations in the lesions created within each treatment group. This variability would greatly affect the eventual functional recovery as measured by the BBB locomotor rating scale. For each animal, lesion cavity was examined in n ≥ 4 coronal sections and averaged. Spared tissue was examined in n = 6 coronal sections and averaged. Each data point refers to one animal.


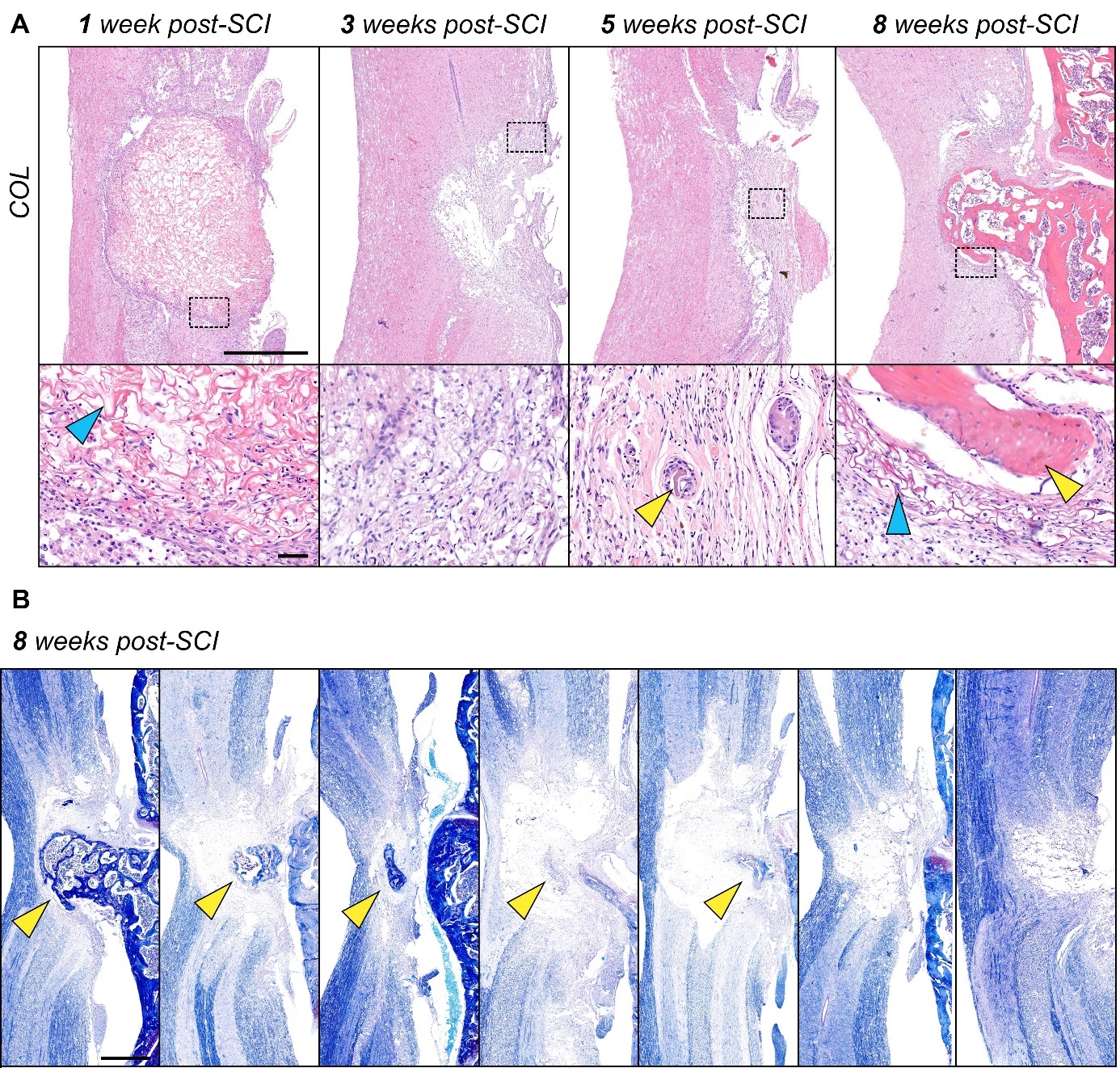


Figure S3. Orthotopic bone formation following implantation of COL-scaffolds. H&E (A) and Klüver-Barrera (B) histological were performed to visualize the orthotopic bone formation throughout the complete experimental period. Note the formation of trabecular bone struts and presence of bone marrow foci after 8 weeks of implantation. Blue arrows depict the COL matrix, and yellow arrows represent bone tissue. Bone tissue was formed in n = 5 out of 7 animals receiving a COL scaffold.


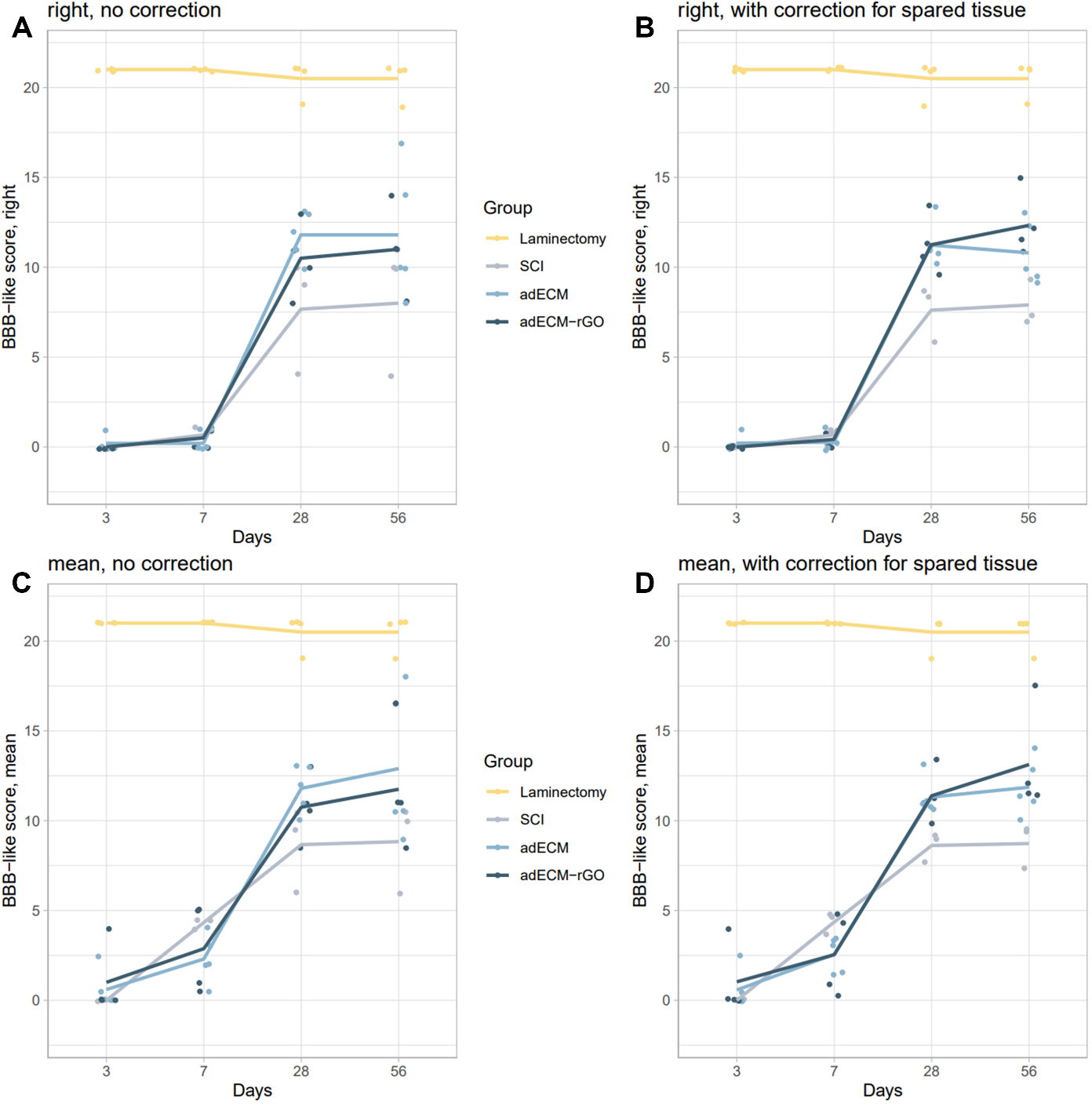
Figure S4. Functional recovery was significantly improved following adECM and adECM-rGO implantation in subgroup analysis. Because of the substantial impact of including ‘Spared Tissue’ into the regression model and given the lesion size drives functional outcomes, a subgroup analysis was performed in which animals that did not show any recovery 1 week post-SCI (BBB-like score of 0, 1, or 2) were included. Recovery of the BBB-like score for the right hindlimb before and after including ‘Spared Tissue’ is presented in Panel A and B, respectively. Note that including ‘Spared Tissue’ into the model led to a smaller dispersion of data points and a shift in the means of all groups, and implantation of both adECM and adECM-rGO led to significantly improved functional recovery. Recovery of the BBB-like score for both hindlimbs (mean) before and after including ‘Spared Tissue’ in Panel C and D, respectively. Here, no significant differences were observed. The number of animals examined for each group throughout the experimental period: laminectomy (n = 4), SCI (n = 3), adECM (n = 5), adECM-rGO (n = 4).

Table S1. Regression analysis results of BBB-like scores of the right hindlimb, without early responders.

| **Without early responders** |  |  |  |  |
| --- | --- | --- | --- | --- |
| **BBB-like score (right)** |  |  |  |  |
|  | estimate | conf.low | conf.high | p.value |
| (Intercept) | -3.283 | -4.982 | -1.584 | 0.001 (***) |
| **Group adECM** | 1.580 | 0.350 | 2.809 | 0.022 (*) |
| **Group adECM-rGO** | 1.950 | 0.646 | 3.254 | 0.008 (**) |
| Spared Tissue | 10.929 | 5.047 | 16.810 | 0.001 (***) |
| Days7 | 0.333 | -1.026 | 1.693 | 0.653 |
| Days28 | 10.250 | 8.891 | 11.609 | <0.001 (***) |
| Days56 | 10.500 | 9.141 | 11.859 | <0.001 (***) |
| **BBB-like score (mean)** |  |  |  |  |
| (Intercept) | -2.197 | -4.368 | -0.026 | 0.104 |
| **GroupadECM** | 1.152 | -0.477 | 2.781 | 0.254 |
| **GroupadECM-rGO** | 1.594 | -0.133 | 3.321 | 0.147 |
| SparedTissue | 9.396 | 1.606 | 17.186 | 0.069 |
| Days7 | 2.417 | 0.766 | 4.067 | 0.008 (**) |
| Days28 | 10.083 | 8.433 | 11.734 | <0.001 (***) |
| Days56 | 10.917 | 9.266 | 12.567 | <0.001 (***) |
